# Left ventricular assist device explantation using a new double-patch technique^†^

**DOI:** 10.1093/icvts/ivad110

**Published:** 2023-07-24

**Authors:** Oliver Daniel Bhadra, Jonas Pausch, Hug Aubin, Payam Akhyari, Artur Lichtenberg, Markus Johannes Barten, Yousuf Alassar, Hermann Reichenspurner, Alexander Markus Bernhardt

**Affiliations:** University Heart & Vascular Center Hamburg, Department for Cardiovascular Surgery, Hamburg, Germany; University Heart & Vascular Center Hamburg, Department for Cardiovascular Surgery, Hamburg, Germany; Heinrich Heine University Duesseldorf, Department for Cardiovascular Surgery, Duesseldorf, Germany; RWTH Aachen University, Department for Cardiovascular Surgery, Aachen, Germany; Heinrich Heine University Duesseldorf, Department for Cardiovascular Surgery, Duesseldorf, Germany; University Heart & Vascular Center Hamburg, Department for Cardiovascular Surgery, Hamburg, Germany; University Heart & Vascular Center Hamburg, Department for Cardiovascular Surgery, Hamburg, Germany; University Heart & Vascular Center Hamburg, Department for Cardiovascular Surgery, Hamburg, Germany; University Heart & Vascular Center Hamburg, Department for Cardiovascular Surgery, Hamburg, Germany

**Keywords:** Left Ventricular Assist Device Explantation, Heart failure, Mechanical Circulatory Support, Left Ventricular Assist Device weaning

## Abstract

**OBJECTIVES:**

There are several surgical approaches for explanting a left ventricular assist device (LVAD) after recovery of cardiac function. Thus, remaining ventricular assist device components may bear significant risks of infection or thrombosis. We hereby report our technique and two-center experience with explantation of LVADs using a new double-patch technique.

**METHODS:**

From March 2019 to April 2021, five patients underwent LVAD explantation after myocardial recovery (HVAD, n = 2; HeartMate 3, n = 3). The mean patient age was 50.3 years (100% male); the mean time on the LVAD was 23.1 ± 20.8 months. The aetiology of the primary heart failure was dilated cardiomyopathy (n = 4) and myocarditis (n = 1).

LVAD explantation was performed using a median sternotomy and cardiopulmonary bypass. The LVAD was stopped, and the outflow graft was clamped. The outflow graft was ligated and sutured close to the aortic anastomosis. The driveline was clipped and removed. Under induced fibrillation, the attachment of the LVAD was released from the apical cuff and the LVAD was removed. A round pericardial patch was fixed from the inner of the ventricle. This step sealed the apex of the heart. An additional Gore-Tex patch was continuously sutured epicardially over the suture ring.

**RESULTS:**

The 5 cases showed technically uncomplicated explantation of the LVADs. During the follow-up of a mean of 16.4 ± 16.9 months, we observed 100% survival. There were no bleeding complications or thromboembolic events during the follow-up period.

**CONCLUSIONS:**

LVAD explantation with the double-patch technique is feasible and safe. This technique allows discontinuation of anticoagulation. The 30-day survival was 100%. Further studies are needed to provide better evidence for LVAD explantation and long-term follow-up.

**Figure:**
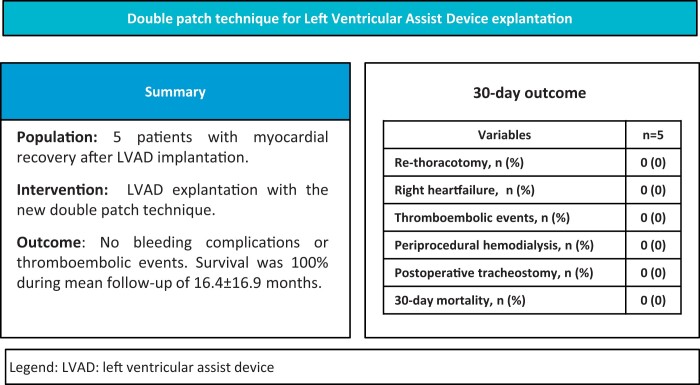

## INTRODUCTION

The clinical indication for a left ventricular assist device (LVAD) implant is progressive heart failure (HF) with severe left ventricular (LV) dysfunction in the setting of exhausted medical therapy [1]. Mechanical circulatory support with an LVAD has become a long-term treatment option for advanced HF. Most patients on an LVAD are either referred for a transplant or are candidates for destination therapy [2, 3]. Unfortunately, only a small proportion of patients can undergo an LVAD explant after recovery of LV function. Overall low recovery rates (1–2%) are reported in the literature [4]. The potential for cardiac recovery depends, in part, on the primary aetiology of the underlying HF. In addition, from a physiological standpoint, LVAD therapy via ventricular unloading is assumed to result in reverse remodelling leading to recovery of LV function [5‒7]. However, little is known about the physiology of LVAD therapy. Explanting an LVAD from a patient with recovered ventricular function presents a surgical challenge. On the one hand, one does not want to jeopardize the haemodynamically and clinically stable patient with an overly complex procedure. On the other hand, too limited a procedure may lead to possible long-term complications. Nevertheless, explanting an LVAD during cardiac recovery seems beneficial to avoid LVAD-related complications [8].

So far, different techniques have been described. Thus, decommissioning with closure of the outflow graft and capping of the driveline is described. In these cases, the LVAD was left in place. With this method, a high infection rate of 41% and high mortality have been described [9].

A retrospective multicentre study analysed the use of a titanium plug to close the apical fixation ring after LVAD explantation and showed a low incidence of plug-related complications [10]. The use of different plug systems with total explantation of the LVAD and driveline was described previously [11]. Another technique involves complete removal of the LVAD and suture ring with double-patch repair of the apex. This highly invasive method may pose a higher risk of LV bleeding and injury in comparison [12]. Retention of the LVAD system and driveline could pose an increased risk of infection and thrombosis. Nevertheless, LVAD explantation seems reasonable in patients with myocardial recovery, although long-term data and comparative studies are lacking. The goal of this study was to perform and investigate a new, easy-to-replicate LVAD explantation technique.

## METHODS

### Ethics statement

Due to the retrospective study design and anonymous data collection, written patient informed consent was waived as considered and approved by our local ethical committee.

### Patients

From March 2019 to April 2021, five patients underwent an LVAD explant after myocardial recovery (HVAD, *n* = 2; HeartMate 3, *n* = 3) at the University Heart & Vascular Center, Hamburg, Germany (*n* = 4), and Heinrich Heine University, Duesseldorf, Germany (*n* = 1). The mean patient age was 50.2 years (100% male); the mean time on the LVAD was 23.1 ± 20.8 months. Primary HF aetiology was dilated cardiomyopathy (*n* = 4) and myocarditis (*n* = 1).

All patients were evaluated for LVAD explantation according to a weaning protocol that included an echocardiographic assessment of the LVEF, which should be >40% with LVAD support, as well as an assessment of heart valve function. In addition, invasive pressures were determined by right heart catheterization. Cardiac output was determined by thermodilution under bicycle ergometric stress and reduction of LVAD speed. Furthermore, patients must not have been hospitalized in the past 6 months and must be receiving optimal guideline-derived HF medication.

### Surgical set-up and technique for the left ventricular assist device explantation

**Figure 1:**
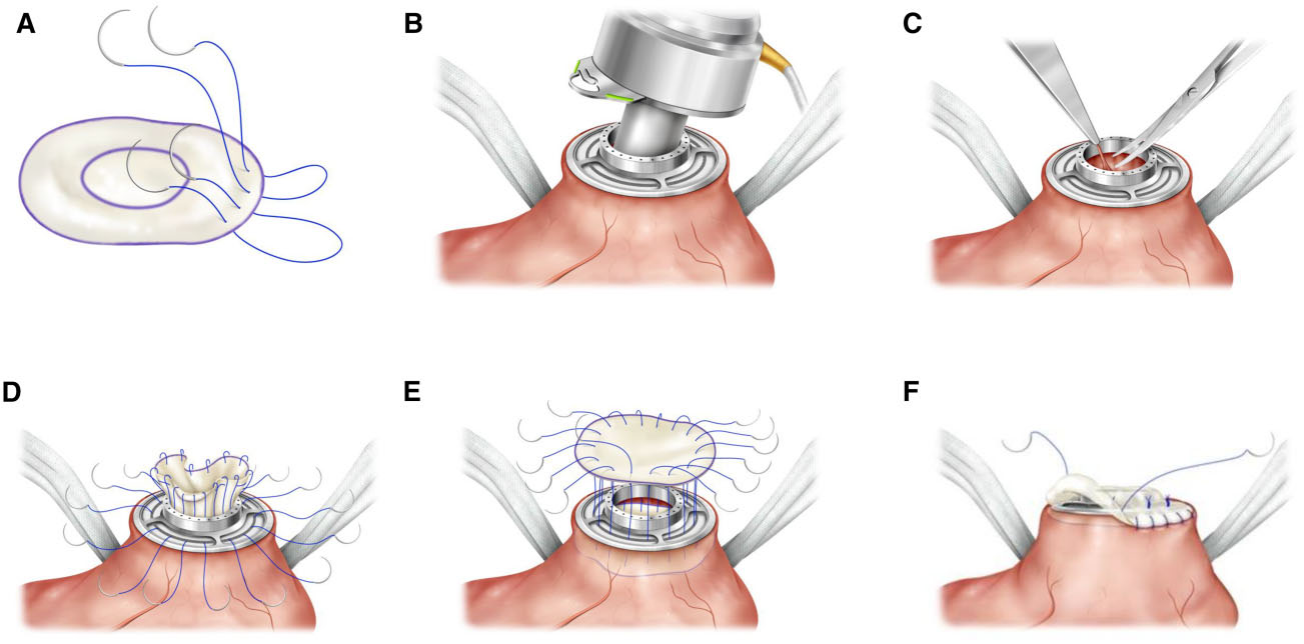
Left ventricular assist device (LVAD) explantation technique: (**A**) A pericardial patch with Prolene sutures was prepared. The pericardial patch is sized to completely cover the suture ring. (**B**) The driveline was clipped and removed. Under induced fibrillation, the attachment of the LVAD was released from the apical suture ring and removed. (**C**) Potential adhesions were excised. (**D**) A round pericardial patch was fixed from the inner edge of the ventricle with twelve 3–0 Prolene sutures stitched from the ventricle towards the suture ring of the LVAD. This procedure seals the apex of the heart. (**E‒F**) An additional Gore-Tex patch is continuously sutured epicardially over the suture ring.

Surgery was performed with the patient under general anaesthesia and on normothermic extracorporeal circulation. LVAD explantation was performed using a median sternotomy. The LVAD was stopped, and the outflow graft was clamped. The outflow graft was ligated and sutured close to the aortic anastomosis. A bovine pericardial patch with Prolene sutures was prepared (Fig. 1A). The diameter of the pericardial patch should be 1 cm larger than that of the suture ring. The Prolene sutures should be sewn 5 mm from the edge of the pericardial patch. The driveline was clipped and removed. Under electrically induced fibrillation, the attachment of the LVAD was released from the apical cuff and the LVAD was removed (Fig. 1B). Potential adhesions were excised (Fig. 1C). A round bovine pericardial patch was fixed from the inner edge of the ventricle with twelve 3–0 Prolene sutures stitched from the ventricle towards the suture ring of the LVAD (Fig 1D). This step sealed the apex of the heart. An additional Gore-Tex patch was continuously sutured epicardially over the suture ring (Fig. 1E‒1F). The ventricle was carefully de-aired. The patient was then weaned from extracorporeal circulation.

### Statistical analysis

Baseline data and perioperative variables were collected in a dedicated institutional LVAD database. Data are presented as means and standard deviation or median and interquartile range for continuous variables and as absolute numbers and percentages for categorical variables. All variables were analysed retrospectively.

## RESULTS

### Patient characteristics

From March 2019 to April 2021, five patients had LVADs explanted after myocardial recovery (HVAD, *n* = 2; HeartMate 3, *n* = 3). The mean patient age was 50.2 years; 100% were male; and the mean time on the LVAD was 23.1 ± 20.8 months. The primary HF aetiologies were dilated cardiomyopathy (*n* = 4) and myocarditis (*n* = 1). Baseline characteristics are shown in Table 1. Before the LVAD was implanted, 80% of the patients were classified as INTERMACS class I or II and 20% as class 3. A total of 60% had short-term mechanical circulatory support before the LVAD was implanted. Furthermore, 60% of patients had chronic renal failure, and 40% were previously on temporary renal replacement therapy.

**Table 1:** Patient characteristics

Variables	n = 5
Age (years)	50.3 ± 8.7
Male sex, n (%)	5 (100)
Arterial hypertension, n (%)	0 (0)
Diabetes mellitus, n (%)	0 (0)
Chronic kidney disease, n (%)	3 (60)
Previous dialysis, n (%)	2 (40)
Pre-LVAD short-term mechanical circulatory support, n (%)	3 (60)
COPD, n (%)	1 (20)
Prior cardiac surgery, n (%)	0 (0)
History of atrial fibrillation, n (%)	3 (60)
Implantable cardioverter defibrillator, n (%)	0 (0)
Aetiology of cardiomyopathy	
Nonischaemic, n (%)	5 (100)
Ischaemic, n (%)	0 (0)
Prior stroke, n (%)	1 (20)
INTERMACS class at time of LVAD implant procedure	
1, n (%)	3 (60)
2, n (%)	1 (20)
3, n (%)	1 (20)
4‒7, n (%)	0 (0)
Device implanted	
Heart Mate III, n (%)	3 (60)
HeartWare, n (%)	2 (40)
Sternotomy for LVAD implant procedure, n (%)	4 (80)

COPD: chronic obstructive pulmonary disease; INTERMACS: Interagency Registry for Mechanically Assisted Circulatory Support; LVAD: left ventricular assist device.

### Procedural characteristics and outcome

The LVAD was explanted via a sternotomy in all cases. The median surgery time was 495 (IQR 110) min, and the median cardiopulmonary bypass time was 219 (IQR 18) min. As a combined procedure, 2 cases of mitral valve repair and 1 case of tricuspid valve repair were performed. A temporary right ventricular assist device implant was not necessary in any case. Results are presented in Table 2. The 5 cases showed technically uncomplicated LVAD explant procedures (Table 3.). In one case, venoarterial extracorporeal membrane oxygenation and an Impella CP implant were required because of intraoperative biventricular function impairment. Mechanical circulatory support was successfully terminated after recovery of LV function after 4 days. Moreover, 30-day survival was 100%. There were no bleeding complications or thromboembolic events during a mean follow-up period of 16.4 ± 16.9 months. This technique allowed discontinuation of anticoagulation in all patients without other indications like atrial fibrillation.

**Table 2:** Descriptive analysis of procedural characteristics

Variables	(n = 5)
Full sternotomy, n (%)	5 (100)
Implantation of vaECMO + Impella CP, n (%)	1 (20)
Additional procedures	
Mitral valve repair, n (%)	2 (40)
Tricuspid valve repair, n (%)	1 (20)
Duration of surgery (min), median (IQR)	495 (110)
Cardiopulmonary bypass time (min), median (IQR)	219 (18)

IQR: interquartile range; vaECMO: veno-arterial extracorporeal membrane oxygenation.

**Table 3:** 30-Day outcome

Variables	n = 5
Re-thoracotomy, n (%)	0 (0)
Right heart failure, n (%)	0 (0)
Thromboembolic events, n (%)	0 (0)
Periprocedural haemodialysis, n (%)	0 (0)
Postoperative tracheostomy, n (%)	0 (0)
30-Day mortality, n (%)	0 (0)

## DISCUSSION

Our data suggest that the double-patch technique provides a feasible and safe technique for LVAD explantation after myocardial recovery. The double-patch technique also facilitates LVAD reimplantation if a patient develops a recurrence of HF after explantation. Moreover, due to the use of bovine pericardial patches, oral anticoagulation can be discontinued. In our study with 5 cases, there were no serious complications or deaths during the mean observation period of 16.4 ± 16.9 months; neither were there any infections or thromboembolic events.

Especially in cases of myocarditis and dilated cardiomyopathies, different LVAD explant techniques have been described. In these cases, it has already been shown that retention of the LVAD, the outflow graft and the driveline represents a significantly increased risk for infectious events [9]. The explantation technique we present did not show any infections after explantation with the double-patch technique.

Alternative explant techniques have already been described in the literature. For example, one technique involves complete removal of the LVAD and the suture ring with patch repair of the apex. This highly invasive method may pose a higher risk of LV bleeding and injury in comparison to the other methods. In addition, reinsertion of an LVAD is no longer as feasible in the case of recurrence of HF [12]. Interventional closure of the outflow graft is a possible alternative therapy for high-risk patients. However, this interventional method can only be performed in noninfected LVADs. In addition, there is a mandatory indication for oral anticoagulation due to the remaining inflow cannula. Thus, there is an increased risk for thrombus formation as well as for infections [13, 14]. In addition, a titanium plug system for simplified removal of the pump with closure of the apex via the suture ring has been described in a large cohort of patients, with good results. This procedure can usually be performed without the use of the heart‒lung machine [10]. To date, however, no CE-certified plug system on the market has been approved, so individual approval must be obtained from local health authorities. The double-patch technique is reproducible and safe and requires only the use of bovine pericardial and Gore-Tex patches. The technique allows subtotal explantation except for the suture ring without the occurrence of infection or thrombus formation during the mean observation period of 16.4 ± 16.9 months. The first cases with this new technique were performed by a median sternotomy. An advantage of the sternotomy approach is that the outflow graft can be ligated and sectioned closer to the aorta. However, explantation by lateral thoracotomy is technically more challenging and may be applied to this technique in the future. Further randomized controlled trials, as well as long-term follow-up, are needed to evaluate the different LVAD explant techniques.

### Limitations

Like all observational studies, retrospective studies can suggest but not definitively prove possible causal relationships. In addition, because we were using a new technique, only a small number of patients without a control group were included. Therefore, only descriptive analyses could be performed. Further prospective randomized studies with a control group would be appreciated.

## CONCLUSION

LVAD explantation with the double-patch technique is feasible and safe. Using a bovine pericardial patch, this technique allows discontinuation of anticoagulation. We had no bleeding or thromboembolic events. Overall, we had 100% survival during a mean follow-up period of 16.4 ± 16.9 months.

## Data Availability

All relevant data are within the manuscript and its Supporting Information files. The raw data are stored at our institute and can be provided by a request to the corresponding editor.

## Abbreviations

HF: Heart failure
LV: Left ventricle
LVAD: Left ventricular assist device
